# Lysosomal storage disorders in nonimmune hydrops fetalis diagnosed by exome sequencing

**DOI:** 10.1186/s13023-025-03851-9

**Published:** 2025-07-10

**Authors:** Mona M. Makhamreh, Kavya Shivashankar, Stephanie M. Rice, Sascha Wodoslawsky, Christina Grant, Rodney McLaren, Seth I. Berger, Huda B. Al-Kouatly

**Affiliations:** 1 Department of Obstetrics and Gynecology, Baylor College of Medicine, Houston, TX USA; 2 Department of Obstetrics and Gynecology, University of Illinois College of Medicine, Chicago, IL USA; 3 Division of Maternal-Fetal Medicine, Department of Obstetrics and Gynecology, Sidney Kimmel Medical College at Thomas Jefferson University, Philadelphia, PA USA; 4 Sidney Kimmel Medical College at Thomas Jefferson University, Philadelphia, PA USA; 5 Center for Genetic Medicine Research and Rare Disease Institute, Children’s National Medical Center, Washington, DC USA

**Keywords:** Exome, Lysosomal storage disorders, Hydrops fetalis

## Abstract

Lysosomal storage disorders (LSD) are a group of inherited metabolic diseases that contribute to nonimmune hydrops fetalis (NIHF). Our objective was to review the pooled exome sequencing (ES) diagnostic yield of LSD in NIHF cases. We expanded our previous meta-analysis and updated our search strategy of prenatal ES studies from 1/1/2000 to 8/1/2024. Cases with LSD gene variants were reviewed. Variants were curated based on the current American College of Medical Genetics and Genomics and ClinGen guidelines. Forty-one ES studies met our inclusion criteria. A total of 207/558 NIHF cases yielded a positive diagnosis by ES. LSD cases represented 27/558 (5%) of all clinically diagnosed NIHF cases that had ES. Rate of LSD among NIHF cases with positive genetic diagnosis by ES was 27/207 (13%). The 27 diagnostic variants and 4 additional variants of uncertain significance (VUS) were identified in 9 different LSD genes. All variants were inherited with a recurrence risk of 25%. Mucopolysaccharidosis type VII (MPS VII) was most prevalent (14/27, 52%). Also, the 4 cases with VUS were identified in the *GUSB* gene. Most cases (21/31, 68%) were isolated NIHF. Hydrops recurrence when reported was present in 75% (15/20) of cases. Consanguinity was reported in 57% (12/21) of cases. In conclusion, 5% of all NIHF cases received a genetic diagnosis of LSD by ES. Thirteen percent of NIHF cases that received a genetic diagnosis by ES were attributable to LSD, with MPS VII being the most prevalent condition. Pairing ES results with enzymatic studies can aid variant interpretation and could have potentially upgraded some of the 4 VUS cases, leading to a higher LSD diagnostic yield. Most cases of LSD presenting as NIHF manifest prenatally as isolated NIHF. High rate of NIHF recurrence and consanguinity highlight importance of genetic counseling and testing for LSD.

**Supplementary Information:**

The online version contains supplementary material available at 10.1186/s13023-025-03851-9.

## Introduction

Hydrops fetalis (HF) is a life-threatening fetal condition defined as an abnormal fluid accumulation in two or more fetal compartments on prenatal ultrasound, including ascites, pleural effusion, generalized skin edema, or pericardial effusion [1]. The occurrence of immune HF has dramatically decreased with Rh(D) immunization [2]. Consequently, nonimmune hydrops fetalis (NIHF) accounts for almost 90% of hydrops cases [3]. NIHF has several possible etiologies, most of which are commonly syndromic, neuromuscular, metabolic, lymphatic, cardiovascular, or hematologic [4, 5]. Cases without a clear etiology are considered idiopathic [6]. Lysosomal storage disorders (LSD) represent one category of inborn errors of metabolism that cause NIHF [7, 8]. LSD are caused by defects in the hydrolysis of macromolecules, the transport of lysosomal metabolites, or the sorting of lysosomal enzymes. These defects result in the accumulation of undegraded material, hindering normal cellular function and triggering a cascade of pathological outcomes [9]. The mechanism by which LSD cause NIHF is not entirely clear but could be secondary to venous obstruction caused by hepatosplenomegaly, heart failure, anemia, or hypoproteinemia [10].

Prenatal diagnosis of LSD can be performed via enzymatic analysis of cultured amniocytes or via molecular genetic testing using a gene panel or exome sequencing (ES) [2, 6, 11]. LSD have been reported to account for 6.6% of causes of NIHF [12]. However, LSD are not typically evaluated in the initial standard-of-care workup for NIHF as defined by Society for Maternal–Fetal Medicine (SMFM) guidelines [1]. Studies have evaluated the significance of LSD as the underlying etiology of NIHF, but precise frequencies have been biased due to cohort selection and literature reporting bias [12, 13]. The objective of this study was to review the pooled ES diagnostic yield of LSD in NIHF cohorts.

## Materials and methods

### Sources

We expanded our previous meta-analysis and systematic literature review from January 1, 2000 to August 1, 2024 [5]. This study was registered under PROSPERO identification tag CRD42022369438 and did not require IRB approval. The search was conducted using the same search terms and databases as previously published [5]. Articles describing cases of NIHF evaluated by prenatal ES were identified. Articles that included cases relevant to both prenatal ES and NIHF were reviewed and the reported variants in a LSD gene were recorded. The reported variants were generally biallelic due to the autosomal recessive nature of these disorders. NIHF was defined as abnormal fluid collection in ≥ 2 fetal compartments (skin edema, ascites, pleural effusion, or pericardial effusion) in the absence of red cell alloimmunization. Only studies that included ≥ 3 cases of NIHF that were evaluated by prenatal ES were included.

### Study selection

We expanded our previous literature search to August 2024, as the prior review had a search end date of December 2021. The final integrated cohort included 27 fetuses with prenatally detected NIHF that were definitively diagnosed with a LSD by ES and an additional 4 possible diagnoses. Supplemental Fig. 1 shows the updated PRISMA flowchart.

**Fig. 1:**
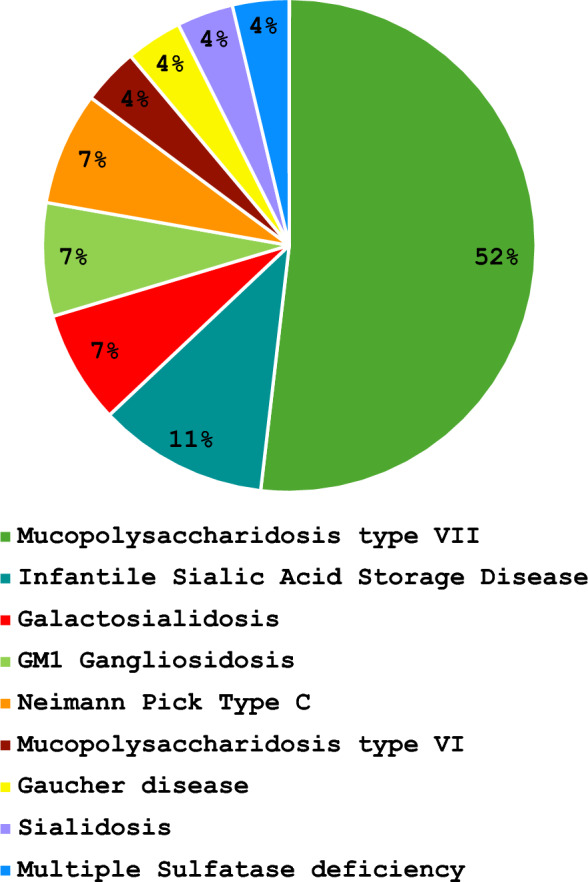
Types of LSD associated with NIHF diagnosed by ES

### Outcomes

Primary outcomes included reported genes, variants, variant interpretations, and inheritance patterns. Secondary outcomes included evaluation of prenatal ultrasound findings.

### Variant classification and interpretation

All variants reported by ES in each NIHF case, including those reported as diagnostic, uncertain, in candidate genes, or unrelated, were recorded from text or supplementary materials. Variants were normalized and processed through a variant validator to ensure they were matched to the MANE select version of the refSeq transcripts database [14, 15]. We harmonized the variant classifications with current American College of Medical Genetics and Genomics (ACMG) and ClinGen guidelines for application of evidence. Notably, we included ClinGen's updated guidelines for use of PVS1 and PP3 criteria. The use of PP5, “report by a reputable source”, criteria required at least two entries in ClinVar to avoid double counting in cases where ClinVar entries were from the cases reported in the paper. NIHF cases that had a variant in LSD genes described as ACMG "pathogenic," "likely pathogenic," or "variants of uncertain significance" (VUS) were extracted. Variant classifications were subsequently reviewed by a board-certified clinical geneticist (S.B.) to ensure strict adherence to ACMG variant classification guidelines [16, 17].

### Quality assessment

Quality assessment was performed using modified standards for reporting of diagnostic accuracy studies (STARD) criteria [18]. The criteria deemed most important to optimize accuracy were: (1) exome sequencing described; (2) use of ACMG criteria for variant interpretation; and (3) definition of hydrops phenotype satisfied.

### Statistical analysis

Statistical heterogeneity was assessed using Higgins *I*^2^ (quantitative) test. A random effects model was conducted to perform a meta-analysis of proportions, comparing the ES diagnostic yield of LSD for NIHF to all cases of NIHF and the proportion of LSD cases diagnosed by ES among all NIHF cases with a positive genetic diagnosis by ES. Diagnostic yield from each study was pooled to estimate diagnostic yield of ES using Metaprop version 8.02 statistical software and 95% confidence intervals (CI) were computed. Results were displayed in forest plots with corresponding 95% CI. Standard error by logit transformed proportion of ES diagnostic yield was performed to evaluate publication bias for LSD in NIHF and for NIHF cases with positive ES diagnosis (Table 1).

**Table 1:** NIHF Cases with LSD Diagnosed by ES

Paper	Gene	Variant	Inheritance	Isolated	Recurrence	Consanguinity	Pregnancy outcome
Shamseldin 2015 [29]	*GUSB*	NM_000181.4:c.307C>T [p.(Arg103Trp)]	Homozygous	Yes	Yes	Yes	Stillbirth
Shamseldin 2015	*GUSB*	NM_000181.4:c.1586A>G [p.(Tyr529Cys)]	Homozygous VUS^†^	Yes	Yes	Yes	Stillbirth
Shamseldin 2015	*GUSB*	NM_000181.4:c.1144C>T [p.(Arg382Cys)]	Homozygous	Yes	Yes	Yes	Stillbirth
Shamseldin 2015	*CTSA*	NM_000308.4:c.595del [p.(Leu199PhefsTer59)]	Homozygous	Yes	Yes	Yes	Stillbirth
Shamseldin 2015	*GUSB*	NM_000181.4:c.398G>C [p.(Trp133Ser)]	Homozygous	Yes	Yes	Yes	Stillbirth
Shamseldin 2015	*GUSB*	NM_000181.4:c.1069C>T [p.(Arg357Ter)]	Homozygous	Yes	Yes	Yes	Stillbirth
Shamseldin 2018 [31]	*GUSB*	NM_000181.4:c.1429C>T [p.(Arg477Trp)]	Homozygous	Yes	No	Yes	TOP or stillbirth
Shamseldin 2018	*CTSA*	NM_000308.4:c.521T>C [p.(Leu174Pro)]	Homozygous	Yes	Yes	Yes	TOP or stillbirth
Shamseldin 2018	*SLC17A5*	NM_012434.5:c.744_747del [p.(Ser249ThrfsTer21)]	Homozygous	Yes	Yes	Yes	TOP or stillbirth
Shamseldin 2018	*SLC17A5*	NM_012434.5:c.1111+1G>A [p.??]	Homozygous	Yes	Yes	Yes	TOP or stillbirth
Shamseldin 2018	*GUSB*	NM_000181.4:c.1429C>T [p.(Arg477Trp)]	Homozygous	Yes	Yes	Yes	TOP or stillbirth
Lefebvre 2020 [39]	*NPC1*	NM_000271.5:c.2819C>T [p.(Ser940Leu)]	Homozygous AR	No	NR	No	Stillbirth 32w
Sparks 2020 [40]	*NPC1*	NM_000271.5:c.3182T>C [p.(Ile1061Thr)] NM_000271.5:c.2072C>A [p.Pro691Gln]	Compound heterozygous AR	No	NR	No	Neonatal death
Sparks 2020	*GLB1*	NM_000404.4:c.931G>A [p.(Gly311Arg)] NM_000404.4:c.75+1delG [p.??]	Compound heterozygous AR	No	NR	No	TOP
Sparks 2020	*GUSB*	NM_000181.4:c.35T>C [p.(Leu12Pro)] NM_000181.4:c.210+1G>A [p.??]	Compound heterozygous AR	No	NR	No	Stillbirth
Sparks 2020	*GUSB*	NM_000181.4:c.1392-10_1476+10del [p.??]	Homozygous AR	Yes	NR	No	Living infant
Al-Kouatly 2021 [41]	*SUMF1*	NM_182760.4:c.691dup [p.(Trp231LeufsTer11)]	Homozygous AR	No	No	No	TOP at 26w
Al-Kouatly 2021	*NEU1*	NM_000434.4:c.238C>A [p.(Pro80Thr)] NM_000434.4:c.1230_1234delinsGCCAAA [p.(Ser410ArgfsTer29)]	Compound Heterozygous AR	Yes	No	No	Neonatal death
Al-Kouatly 2021	*GUSB*	NM_000181.4:c.1084G>A [p.(Asp362Asn)] NM_000181.4:c.1747G>A [p.(Gly583Arg)]	Compound heterozygous AR	No	No	No	Stillbirth 27w
Zhou 2021 [30]	*GBA*	NM_000157.4:c.1448T>C [p.(Leu483Pro)]	Homozygous	Yes	Yes	NR	TOP at 26w
Zhou 2021	*GUSB*	NM_000181.4:c.1192C>T [p.(Arg398Cys)]	Homozygous VUS^†^	No	Yes	NR	TOP at 27w
Zhou 2021	*GUSB*	NM_000181.4:c.104C>A [p.(Ser35Ter)] NM_000181.4:c.1091C>T [p.Pro364Leu]	Compound heterozygous	Yes	Yes	NR	TOP
Zhou 2021	*GUSB*	NM_000181.4:c.1610T>C [p.(Ile537Thr)] NM_000181.4:c.323C>T [p.(Ile410Arg)]	Compound heterozygous VUS^†^	No	Yes	NR	Neonatal death
Correa 2021 [42]	*GUSB*	NM_000181.4:c.1729C>T [p.(Arg577Cys)]	Homozygous	No	Yes	Yes	NR
Chen 2022 [19]	*GUSB*	NM_000181.4:c.1324G>A [p.Ala442Thr]	Homozygous VUS^†^	Yes	NR	NR	NR
Dufke 2022 [20]	*GLB1*	NM_000404.4:c.808T>G [p.Tyr270Asp] NM_000404.4:c.699del [p.Gln234Argfs*20]	Compound heterozygous	Yes	NR	NR	NR
Dufke 2022	*ARSB*	NM_000046.5:c.1577del [p.Thr526Metfs*48]	Homozygous	Yes	NR	NR	NR
Marangoni 2022 [21]	*SLC17A5*	NM_012434.5:c.308G>A [p.Trp103*] NM_012434.5: dup ex 8–9	Compound heterozygous	No	NR	NR	TOP at 25w
Allen 2023 [25]	*GUSB*	NR*	NR	Yes	NR	NR	Neonatal death
Allen 2023	*GUSB*	NR*	NR	Yes	NR	NR	Live birth
Tran Mau-Them 2023 [28]	*GUSB*	NM_000181.3:c.526C>T [p.(Leu176Phe)] NM_000181.3:c.1145G>A [p.(Arg382His)]	Compound heterozygous	Yes	No	No	TOP

*ES* exome sequencing, *NIHF* nonimmune hydrops fetalis, *NR* not reported, *TOP* termination of pregnancy, *VUS* variant of uncertain significance

*Variants were not available and could not be validated

^†^Variants were reclassified from pathogenic/likely pathogenic to VUS based on harmonized variant classification criteria

## Results

### Study and demographic characteristics

A total of 41 studies reporting 558 pregnancies with NIHF met our inclusion criteria. Ten additional studies were found with the updated search dates after our prior meta-analysis [19–28]. Of the 41 studies, 12 studies reported a total of 31 pregnancies with prenatal NIHF due to LSD. Of the 27 pregnancies with reported pregnancy outcomes, there were nine (9/27, 33%) stillbirths, seven (7/27, 26%) pregnancy terminations, and six livebirths (6/27, 22%), of which four (4/6, 67%) were neonatal deaths. Five pregnancies (5/27, 19%) were reported in aggregate as either terminations or stillbirths. Pregnancy outcome was not reported in four (4/31, 13%) cases.

Isolated versus non-isolated NIHF phenotype, consanguinity, and recurrence were reviewed. Twenty-one LSD-positive NIHF cases (21/31, 68%) were isolated, while 10 were non-isolated (10/31, 32%) with additional findings on prenatal imaging (Table 2). These prenatal abnormalities were as follows: Hepatomegaly and/or splenomegaly (4/10, 40%), renal (4/10, 40%), and clubbed feet (3/10, 30%). Consanguinity was addressed in 21 cases, with a total of 12 cases reported (12/21, 57%). Of the 20 pregnancies where recurrence history was reported, 15 cases (15/20, 75%) reported recurrence of HF in other pregnancies.

**Table 2:** LSD genes and prenatal phenotype in non-isolated NIHF

Paper	Gene	Ultrasound findings in addition to NIHF
Lefebvre 2020	*NPC1*	Hepatic cirrhosis Autopsy: Ascites, hepatic fibrosis, splenomegaly
Sparks 2020	*NPC1*	Biventricular cardiac hypertrophy, placentomegaly
Sparks 2020	*GLB1*	Thick NF, VSD, pelvic kidney, SUA
Sparks 2020	*GUSB*	Thick NF, unilateral multicystic kidney disease, oligohydramnios
Al-Kouatly 2021	*SUMF1*	Echogenic kidneys
Al-Kouatly 2021	*GUSB*	Hypertelorism, hepatomegaly, left renal pyelectasis, bilateral clubbed feet
Zhou 2021	*GUSB*	Clubbed feet, NT = 1.9 mm
Zhou 2021	*GUSB*	Clubbed feet, NT = 1.7 mm
Correa 2021	*GUSB*	Coarse face, hepatosplenomegaly, bony changes, contractures
Marangoni 2022	*SLC17A5*	Hepatomegaly, echogenic kidneys

*LSD* lysosomal storage disorder, *NF* nuchal fold, *NT* nuchal translucency, *NIHF* nonimmune hydrops fetalis, *SUA* single umbilical artery, *VSD* ventricular septal defect

### Diagnostic genetic testing

Among the 41 ES studies, 207 of 558 NIHF cases received a genetic diagnosis by ES. Of all NIHF cases with a positive genetic diagnosis by ES, 27/207 (13%) were due to LSD. Among all cases that underwent ES, cases with LSD represented 27/558 (5%) of all NIHF cases. The four cases with VUS were not included in these calculations. ES was maternal-paternal-fetal trio in 15 cases (15/31, 48%), fetal only in 13 cases (13/31, 42%), and three cases did not specify the type of ES performed (3/31, 10%).

ES yielded diagnostic molecular variants in nine LSD genes. The LSD genes associated with NIHF diagnosed by ES were as follows: *GUSB* (14/27, 52%), *SLC17A5* (3/27,11%), *CTSA* (2/27, 7%), *GLB1* (2/27, 7%), *NPC1* (2/27, 7%), *ARSB* (1/27, 4%), *GBA* (1/27, 4%), *NEU1* (1/27, 4%), and *SUMF1* (1/27, 4%). Four *GUSB* variants (4/31, 13%) were reclassified from Likely Pathogenic to VUS (Table 1) [19, 29, 30]. Mucopolysaccharidosis (MPS) type VII was the most common LSD (14/27, 52%) (Fig. 1). Variant zygosity was reported as compound heterozygous in 10 cases (10/31, 32%) and homozygous in 19 cases (19/31, 61%). Two cases from one study did not report variant zygosity [25]. All variants where segregation was accomplished with parental ES were inherited (Table 3).

**Table 3:** LSD genes associated with NIHF diagnosed by ES

LSD	Gene	Frequency (N = 27)	Inheritance
Mucopolysaccharidosis, Type VII	*GUSB*	14 (52%)	7 Homozygous 5 Compound Heterozygous 2 NR
Infantile Sialic Acid Storage Disease	*SLC17A5*	3 (11%)	2 Homozygous 1 Compound Heterozygous
Galactosialidosis	*CTSA*	2 (7%)	2 Homozygous
GM1 Gangliosidosis	*GLB1*	2 (7%)	2 Compound Heterozygous
Neiman Pick Disease, Type C	*NPC1*	2 (7%)	1 Homozygous 1 Compound Heterozygous
Mucopolysaccharidosis, Type VI	*ARSB*	1 (4%)	Homozygous
Gaucher	*GBA*	1 (4%)	Homozygous
Sialidosis	*NEU1*	1 (4%)	Compound Heterozygous
Multiple Sulfatase Deficiency	*SUMF1*	1 (4%)	Homozygous

*LSD* lysosomal storage disorders, *NIHF* nonimmune hydrops fetalis, *NR* not reported

*Four *GUSB* VUS were not included in this table

### Systematic review and meta-analysis

**Fig. 2:**
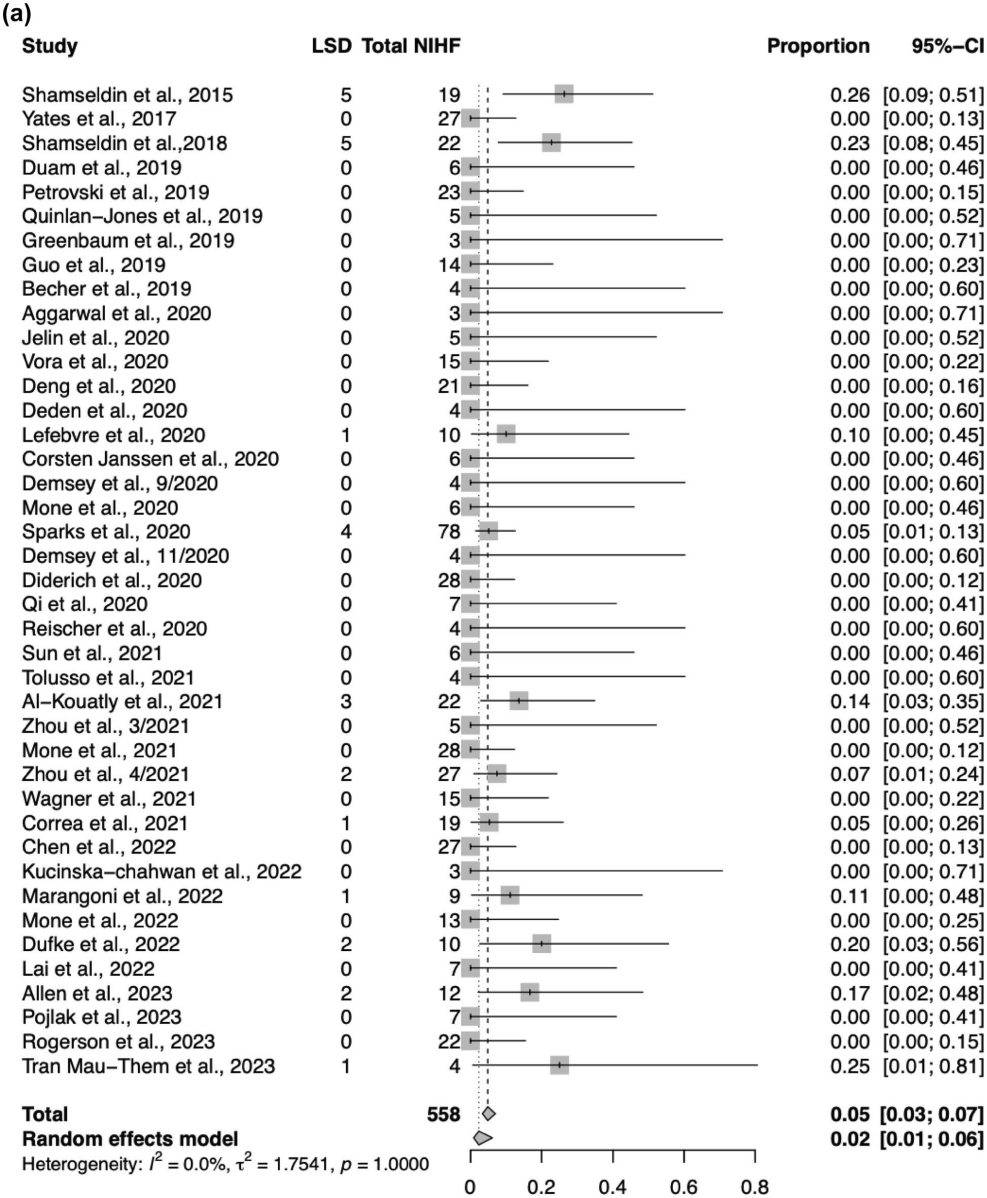

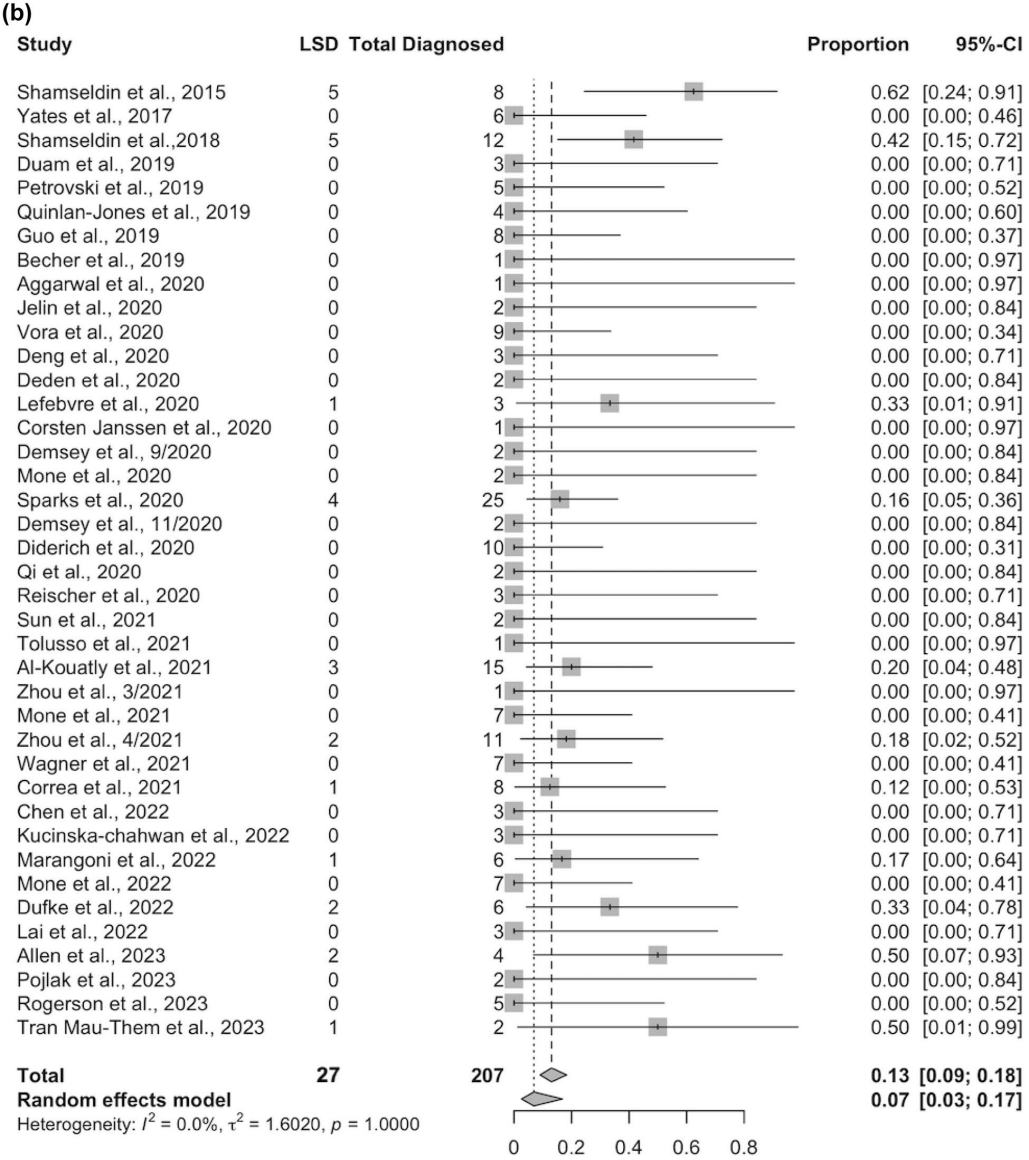
**a** Forest plot demonstrating ES diagnostic yield for LSD in NIHF. **b** Forest plot demonstrating proportion of LSD among all NIHF cases with positive ES diagnosis

**Fig. 3:**
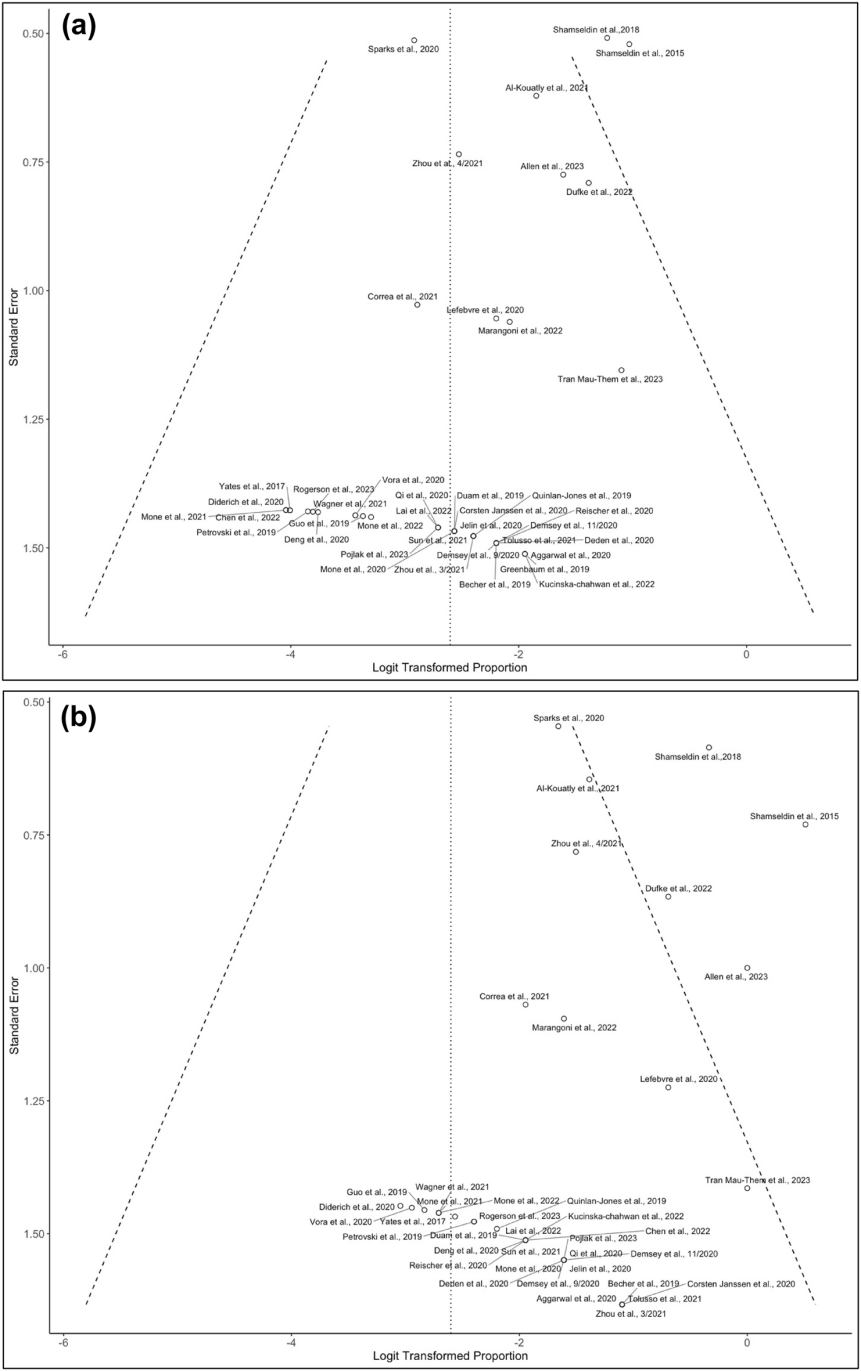
**a** Funnel plot of standard error by logit transformed proportion of ES diagnostic yield to evaluate publication bias for LSD in NIHF. **b** Funnel plot of standard error by logit transformed proportion of LSD to evaluate publication bias for NIHF cases with positive ES diagnosis

The pooled ES diagnostic yield for LSD in NIHF was 5% (95% CI 3–7%; *p* = 1.0; *I*^2^ = 0%) (Fig. 2a). The proportion of LSD among all NIHF cases with an ES diagnosis was 13% (95% CI 9–18%; *p* = 1.0; *I*^2^ = 0%) (Fig. 2b). Standard error by logit transformed proportion of ES diagnostic yield was performed to evaluate publication bias for LSD in NIHF (Fig. 3a). Standard error by logit transformed proportion of LSD to evaluate publication bias for NIHF cases with positive ES diagnosis was also performed (Fig. 3b). Quality assessment is demonstrated in diagram in Supplemental Fig. 2. Two studies by Shamseldin et al. had significantly higher overall diagnostic rate of LSD and this was attributed to cases selected for consanguineous families, which favor diagnosis of recessive disorders [29, 31].

## Discussion

In our meta-analysis, at least 13% of NIHF cases with a positive genetic diagnosis were attributable to LSD. We identified LSD as the second most frequent monogenic disease category associated with NIHF through ES, following RASopathies [32]. Of 558 clinically diagnosed NIHF cases, ES diagnostic yield for LSD was 5%. Excluding the two studies enriched by consanguineous families, the diagnostic yield for LSD was 3% [29, 31]. Twenty-seven diagnostic variants and four VUS were identified in nine different LSD genes. The four VUS were all found in the *GUSB* gene. Among these LSD, MPS VII (*GUSB*) was the most prevalent condition, representing almost half the diagnosed LSD. While the four additional cases of MPS VII were considered diagnosed by the primary authors, our harmonized variant classification downgraded these four cases to VUS in the *GUSB* gene. Therefore, our review emphasizes the value of pairing ES results with enzymatic assays to improve the interpretation of genetic variants. Enzymatic assays could have potentially upgraded some of these four VUS cases with *GUSB* variants, making *GUSB* the third most common single gene associated with NIHF diagnosed by ES after the *PIEZO1* gene and *PTPN11* gene [32, 33].

The 57% fraction of diagnosed cases with reported consanguinity and the 75% fraction of diagnosed cases with reported recurrence of HF was remarkable and consistent with the heightened risk for recessive disorders suggested by these covariates. On further review of our data, overall LSD represented 13.4% (15/112) of recurrent NIHF cases compared to only to 2.3% (9/395) for NIHF cases not reported as recurrent (OR 6.6, CI 2.8–15.6, p < 0.0001). Given that the current data show that LSD are the most common recessive form of NIHF, recurrent NIHF is a strong indicator to consider prenatal enzymatic LSD in addition to molecular testing [7, 34]. This consideration is important when sequencing reveals in a LSD gene the following: (a) Only one pathogenic/likely pathogenic (P/LP) variant; (b) One P/LP variant and one VUS; or (c) Two VUS.

**Fig. 4:**
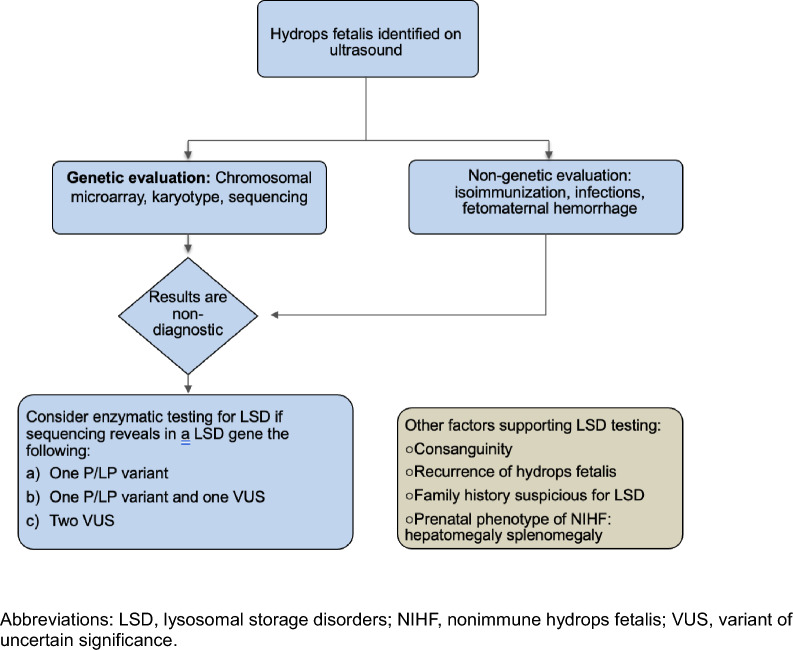
Diagnostic workflow for NIHF cases suspicious for LSD

The association of LSD with NIHF has been well studied over the past several years [9, 12, 13, 35]. While our study significantly revises previous estimates, LSD have been reported to account for 8.2% of causes of idiopathic NIHF [12]. In one review that included 22 LSD case series in 2678 cases of NIHF, the overall incidence of LSD was 6.6% in NIHF cases that were biochemically tested for any LSD. However, neither biochemical nor molecular LSD testing are part of the recommended SMFM initial standard of care work up [1]. The guidelines address the risk for LSD in cases of NIHF when standard work up is negative. Our study further supports the recommendation for incorporating biochemical and molecular testing for LSD to the standard workup of NIHF. Also, our data strongly supports prenatal biochemical testing for LSD, especially in cases of recurrent NIHF. Given the relatively high incidence, and emerging treatment implications including prenatal enzyme replacement therapy, LSD testing is reasonable in the setting of NIHF with negative standard of care work up [12]. The authors created a diagnostic workflow diagram to assist providers in caring for patients with pregnancies complicated by NIHF with high index of suspicion for LSD as an etiology, Fig. 4.

Our results highlight the poor pregnancy outcomes of NIHF cases with a LSD. When reported, 13/15 (87%) continued pregnancies resulted in stillbirth or neonatal death. This finding underscores the importance of establishing a prenatal diagnosis by ES as it can guide pregnancy counseling and targeted management. Additionally, multiple in utero treatments are emerging for specific genetic disorders. There is currently an ongoing phase 1 clinical trial for in utero enzyme replacement therapy for nine LSD, including MPS types I, II, IVA, VI, and VII, Gaucher Type 2, Gaucher Type 3, Pompe disease infantile onset, and Wolman disease (clinicaltrials.gov accessed 4/18/2024) [36]. This trial includes MPS type VII, the most common LSD type diagnosed by ES in cases of NIHF in our review. Approved postnatal therapies for MPS type VII and several other types of LSD include enzyme replacement therapy or substrate reduction therapy that can be initiated without diagnostic delay in children with prenatal diagnoses [12].

Prenatal diagnosis of LSD can be performed on amniotic fluid supernatant or cultured amniocytes obtained by amniocentesis [13]. Testing for LSD can be done by comprehensive LSD enzyme analysis, a LSD gene panel, or ES. The most sensitive method for evaluating LSD is through biochemical evaluation of the amniocytes. Molecular studies can miss LSD diagnosis due to challenging variant classification, particularly in groups underrepresented in reference genomic databases. Thus, biochemical testing for LSD can prove important as orthogonal testing to broad molecular testing for multiple etiologies for hydrops. If we were able to pair enzymatic testing results with the 4 VUS cases, the variant classification could have been upgraded for some variants. The availability of a molecular diagnosis allows future pregnancy planning and can aid pre-implantation genetic testing for monogenic disorders (PGT-M). Identifying the metabolic defect causing NIHF facilitates genetic counseling on recurrence risks and carrier testing to family members, which provides more options for families about potential treatment and reproductive planning.

Most LSD cases reported in this systematic review were isolated NIHF. A retrospective case–control study by our group comparing LSD-positive NIHF cases to LSD-negative NIHF found that NIHF cases diagnosed with LSD were more likely to have hepatomegaly and splenomegaly on prenatal ultrasound [11]. In the current review, it could be that most LSD cases were isolated NIHF due to either lack of prenatal phenotyping of these features in standard Maternal fetal medicine (MFM) practices leading to under reporting of hepatomegaly and splenomegaly, or due to differences in the ascertainment of these populations namely individuals with a high pretest probability of undergoing enzyme testing versus more non-specific presentations undergoing exome sequencing. Thus, such prenatal phenotype in NIHF should prompt an LSD work up, if not already done. Measuring the liver length in NIHF is not standard practice by MFM providers, but it should be considered. The study by Tongprasert et al. evaluates the fetal liver length by gestational age [37]. Data on the reporting of placentomegaly as an ultrasound finding in NIHF due to LSD remains limited based on our review. Only one case reported placentomegaly; the remaining cases did not delineate the presence of normal placenta or placentomegaly.

Maternal complications associated with hydrops fetalis include mirror syndrome. A recent systematic literature review suggests that mirror syndrome falls within the spectrum of preeclampsia [38]. Among the included manuscripts of NIHF due to LSD in this review, none reported on the presence or absence of mirror syndrome during pregnancy.

### Strengths and limitations

To our knowledge, our study is the most comprehensive systematic literature review of NIHF caused by LSD variants diagnosed by ES. We applied a classification strategy to the included cases to ensure uniform assessment of diagnosis across our dataset. This study also described the prenatal phenotype of LSD variants causing NIHF, rather than the phenotype in the postnatal period. Our study was limited by the publication bias inherent to systematic reviews, although we attempted to limit publication bias with our inclusion criterion of studies reporting ≥ 3 NIHF cases. A possible limitation for the spectrum of LSD in NIHF diagnosed by ES could be that we excluded studies with < 3 cases. However, when we reviewed studies that had one or two cases of NIHF with ES, none had LSD as a diagnosis. Another limitation of our study is that we did not have access to the raw data where reanalysis could identify diagnostic variants that were not originally reported due to variant classification criteria at the time of publication. As such, we view our estimated diagnostic yields as lower bounds since additional unreported variants in these cases would only increase the number of diagnoses.

## Conclusions

In conclusion, 5% of all NIHF cases received a genetic diagnosis of LSD by ES. At least 13% of NIHF cases that received a genetic diagnosis by ES were due to LSD with MPS type VII being most prevalent. When LSD presents as NIHF, most cases manifest prenatally as isolated NIHF without associated structural fetal anomalies.

The data that support the findings of this study are available from the corresponding author upon reasonable request.

## Supplementary Information

Additional file 1.

## Data Availability

All data supporting the findings of this study are available within the paper and its Supplementary Information.

## Declarations

### PROSPERO registration number

CRD42022369438.

### Previous presentation

Accepted as a poster presentation at the Society of Maternal Fetal Medicine 43rd Annual Pregnancy Meeting, San Francisco, February 2023.
