# Supplementary material for: Lysosomal storage disorders in nonimmune hydrops fetalis diagnosed by exome sequencing
Source: Orphanet J Rare Dis. 2025 Jul 10;20:351. doi: 10.1186/s13023-025-03851-9 (PMC12243238; doi:10.1186/s13023-025-03851-9)
Supplement: Supplementary file 1 — Additional file 1. [file 13023_2025_3851_MOESM1_ESM.docx]

Supplemental Figure 1. PRISMA Flow Chart

Additional records identified through references

(n = 123)

Records identified through database search

(n = 9,796)

Exclusion of **33** studies:

**25** prenatal ES studies with less than 3 cases of NIHF

**3** studies reporting cases duplicated in a subsequent publication^a,b,c^

**3** studies reported only positive diagnostic results^d,e,f^

**1** study without total number of NIHF cases^g^

**1** study reported results in aggregate and authors not able to obtain raw data^h^

Studies included in

quantitative synthesis

(meta-analysis)

(n = **41**)

Total records screened

(n = 8,308)

Records after duplicates removed (n = 8,308)

Full-text articles assessed

for eligibility

(n = 74)

Exclusion of **8,234** studies:

**252** reviews

**433** case reports

**102** editorial/ comment/ guideline

**490** foreign languages

**151** qualitative or ethical studies

**5,948** not relevant to

prenatal ES

**858** prenatal ES without

NIHF cases

Total duplicates

removed (n = 1,611)

**Identification**

**Screening**

**Eligibility**

**Included**

^a^Vora NL, et al. Prenatal exome sequencing in anomalous fetuses: new opportunities and challenges. Genet Med. 2017 Nov;19(11):1207-1216. doi: 10.1038/gim.2017.33.

^b^Mellis, et al. Diagnostic Yield of Exome Sequencing for Prenatal Diagnosis of fetal structural anomalies: A Systematic Review and Meta‐Analysis. *Prenatal Diagnosis*, vol. 42, no. 6, 2022, pp. 662–685., doi:10.1002/pd.6115.

^c^Wei, et al.The Value of Exome Sequencing in Thoracoamniotic Shunt for Severe Pleural Effusion with Fetal Hydrops: A Retrospective Clinical Study. Fetal Diagn Ther. 2022;49(3):138-144. doi: 10.1159/000521212.

^d^Swanson K, et al. The utility of pathologic examination and comprehensive phenotyping for accurate diagnosis with perinatal exome sequencing. *Prenatal diagnosis*, 2022;42(10), 1288–1294. doi.org/10.1002/pd.6197

^e^Wu W, et al. Outcome and etiology of fetal pleural effusion, fetal ascites, and hydrops fetalis after fetal intervention: retrospective observational cohort from a single institution. *Ultrasound in obstetrics & gynecology, 2023.* doi.org/10.1002/uog.27501.

^f^Bourgon N, et al. Same performance of exome sequencing before and after fetal autopsy for congenital abnormalities: toward a paradigm shift in prenatal diagnosis? *European journal of human genetics*, 2022; 30(8), 967–975. doi.org/10.1038/s41431-022-01117-7

^g^Gabriel, et al. “Trio Exome Sequencing Is Highly Relevant in Prenatal Diagnostics.” Prenatal Diagnosis, vol. 42, no. 7, 2022, pp. 845–51, doi:10.1002/pd.6081

^h^Wei X, et al. An investigation of the etiologies of Non-immune hydrops fetalis in the era of next generation sequence-A single center experience. Genes, 2022;13 (12), doi.org/10.3390/genes13122231

Studies pertaining to LSD

(n=12)

Supplemental Figure 2. Quality assessment of 41 studies included in our systematic review using modified standards for reporting of diagnostic accuracy studies (STARD) criteria.


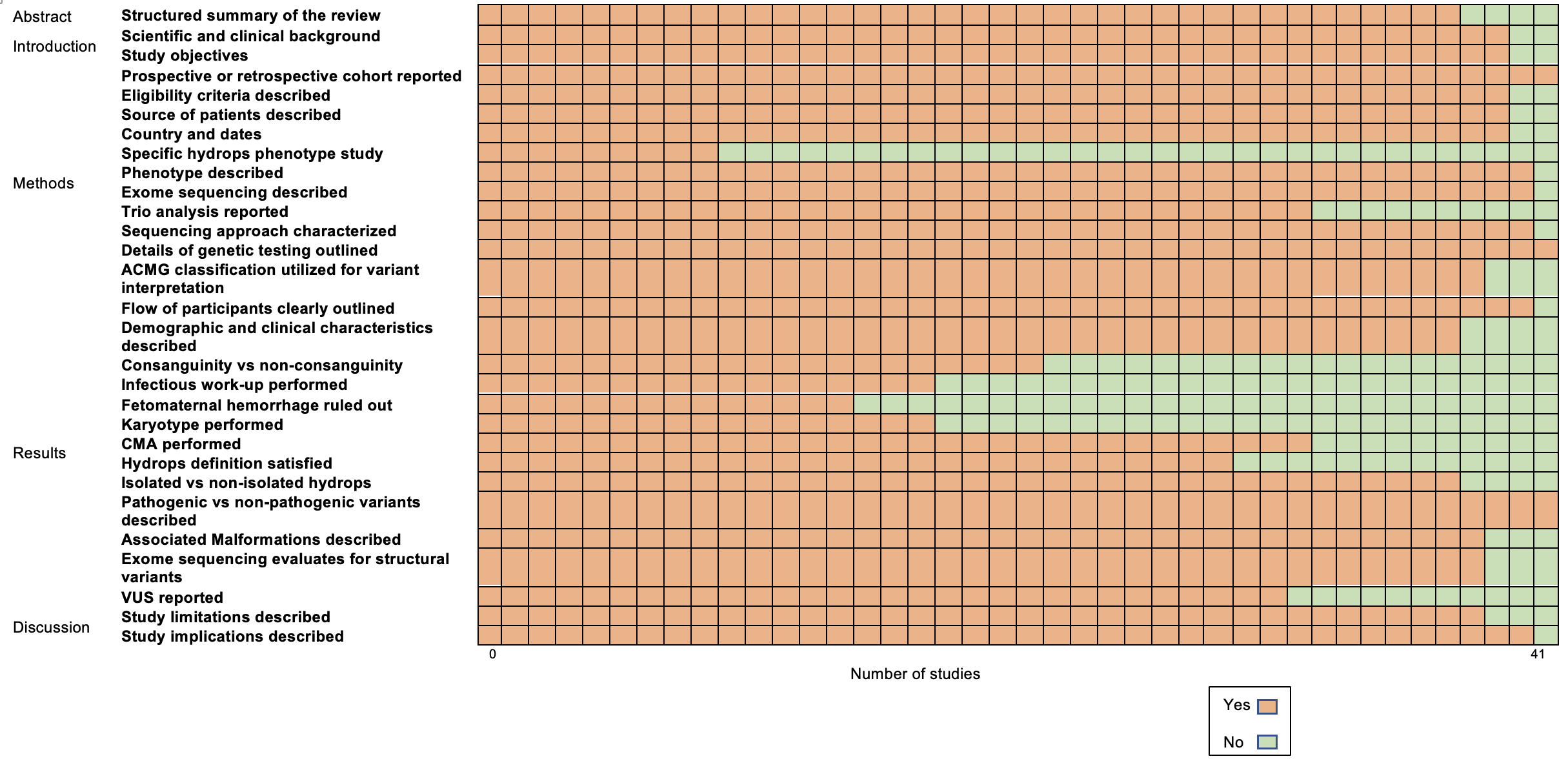


ACMG, American College of Medical Genetics and Genomics; CMA, chromosomal microarray; ES, exome sequencing; VUS, variants of uncertain significance.
